# Role of caveolin-1 as a biomarker for radiation resistance and tumor aggression in lung cancer

**DOI:** 10.1371/journal.pone.0258951

**Published:** 2021-11-11

**Authors:** Dominic Leiser, Santanu Samanta, John Eley, Josh Strauss, Michael Creed, Tami Kingsbury, Paul N. Staats, Binny Bhandary, Minjie Chen, Tijana Dukic, Sanjit Roy, Javed Mahmood, Zeljko Vujaskovic, Hem D. Shukla

**Affiliations:** 1 Division of Translational Radiation Sciences (DTRS), Department of Radiation Oncology, University of Maryland School of Medicine, Baltimore, MD, United States of America; 2 Department of Radiation Oncology, School of Medicine, Vanderbilt University, Nashville, TN, United States of America; 3 Department of Physiology, University of Maryland School of Medicine, Baltimore, MD, United States of America; 4 Department of Pathology, University of Maryland, School of Medicine, Baltimore, MD, United States of America; Virginia Commonwealth University, UNITED STATES

## Abstract

Radiation therapy plays a major role in the treatment of lung cancer patients. However, cancer cells develop resistance to radiation. Tumor radioresistance is a complex multifactorial mechanism which may be dependent on DNA damage and repair, hypoxic conditions inside tumor microenvironment, and the clonal selection of radioresistant cells from the heterogeneous tumor site, and it is a major cause of treatment failure in non–small cell lung cancer (NSCLC). In the present investigation caveolin-1 (*CAV-1*) has been observed to be highly expressed in radiation resistant A549 lung cancer cells. CRISPR-Cas9 knockout of *CAV-1* reverted the cells to a radio sensitive phenotype. In addition, *CAV-1* overexpression in parental A549 cells, led to radiation resistance. Further, gene expression analysis of A549 parental, radiation resistant, and caveolin-1 overexpressed cells, exhibited overexpression of DNA repair genes RAD51B, RAD18, SOX2 cancer stem cell marker, MMPs, mucins and cytoskeleton proteins in resistant and caveolin-1 over expressed A549 cells, as compared to parental A549 cells. Bioinformatic analysis shows upregulation of BRCA1, Nuclear Excision DNA repair, TGFB and JAK/STAT signaling pathways in radioresistant and caveolin-1 overexpressed cells, which may functionally mediate radiation resistance. Immunohistochemistry data demonstrated heterogeneous expression of *CAV-1* gene in human lung cancer tissues, which was analogous to its enhanced expression in human lung cancer cell line model and mouse orthotopic xenograft lung cancer model. Also, TCGA PanCancer clinical studies have demonstrated amplification, deletions and missense mutation in *CAV-1* gene in lung cancer patients, and that *CAV-1* alteration has been linked to poor prognosis, and poor survival in lung cancer patients. Interestingly, we have also optimized ELISA assay to measure caveolin-1 protein in the blood of A549 radiation resistant human xenograft preclinical mouse model and discovered higher level of caveolin-1 (950 pg/ml) in tumor bearing animals treated with radiation, as compared to xenograft with radiosensitive lung cancer cells (450 pg/ml). Thus, we conclude that caveolin-1 is involved in radio-resistance and contributes to tumor aggression, and it has potential to be used as prognostic biomarker for radiation treatment response, and tumor progression for precision medicine in lung cancer patients.

## Introduction

Lung cancer is the second most common cancer in the United States accounting for approximately 13% of all new cancers are lung cancer cases. In 2020, the new cases for lung cancer cases in the United States are estimated to be 228, 820 and about 135,720 deaths [1, 2]. The overall, 5-year survival rates for non-small cell lung cancer (NSCLC) and small cell lung cancer (SCLC) is about 16% [3]. Lung cancer can be treated with surgery, chemotherapy, radiation therapy and immunotherapy, and depending on the stage and clinical circumstance one, two, or three modalities are utilized. In the case of early-stage lung cancer, radiation therapy offers control rates similar to resection alone. For locally advanced NSCLC, where resection may not be feasible, radiation therapy (along with chemotherapy) are the mainstay of treatment. Despite maximal treatment with radiation therapy, NSCLC often recurs. In this setting, relapsed NSCLC are frequently re-irradiated, with poor outcomes and as a result, patients may develop acquired radio resistance [4–7]. Furthermore, cancer cell heterogeneity also plays a significant role in acquired radioresistance [8]. Consequently, it is one of the foremost challenges to develop effective therapeutic options to overcome radio-resistance in lung cancer treatment. To mitigate radio-resistance and improve the survival of lung cancer patients, basic molecular mechanisms responsible for resistance to radiation therapy must be thoroughly investigated. Therefore, novel strategies are required to minimize radioresistance in lung cancer cells.

An inherent challenge in radiation oncology is cancer cells develop resistance to recommended radiation doses leading to cancer relapse. The fundamental mechanisms of radio-resistance are not completely established, and multiple pathways appear to contribute to resistance [5, 9]. Radioresistance can be mediated, by enhanced activation of ATM and RAD related ATR kinases, and two downstream checkpoint kinases Chk1 and Chk2 which have been implicated in almost 7% of lung adenocarcinoma [10–12]. Expansion of cancer stem cells during radiation therapy, and activation of EGFR and PI3K-AKT pathway has also been identified as an inherent radio-resistance mechanism [13–15]. A hypoxic tumor microenvironment has also been linked to radio-resistance [16]. Based on our current investigation caveolin-1 plays an immensely important role in radio-resistance and tumor aggression. caveolin-1 is a 21 kDa membrane protein enriched in caveolae and functionally associated with endocytosis, extracellular matrix organization, cell migration, cancer signaling and radio-resistance in other malignancies [17–20]. The *CAV-1* is highly expressed in many tumors and executes an important role in tumor progression, and with a potential to transform the tumor microenvironment (TME) leading to poor treatment outcome [21, 22]. *CAV-1* has also been implicated in navigating cell migration in stroma and cancer invasion [23, 24].

In the present study, we investigated the molecular basis of radio-resistance in lung cancer A549 cells by knocking down caveolin-1 gene employing CRISPR-Cas9 gene editing technology. We have also investigated overexpression of caveolin-1 in A549 cells and studied its phenotypic consequences. Further, comparative genomic analysis of A549 parental, resistant and overexpressed A549 cells was also performed to identify caveolin-1 activated radio-resistance pathway. We also analyzed TCGA PanCan lung cancer patient’s data using cBioPortal and investigated role of CAV-1 mutation in clinical outcomes. Subsequently, an attempt has been made to corroborate our cell line data with clinical data and optimize ELISA assay to measure caveolin-1 in the blood.

## Materials and methods

### Cell lines and culture conditions

A549 lung cancer cells were cultured and maintained in DMEM medium with 10% FBS, 100 U/ mL penicillin and 2 mM L-glutamine in a humidified atmosphere in an incubator maintained at 37°C. For the experiments, confluent monolayer cultures were trypsinized using 0.25% Trypsin/EDTA and centrifuged at 1000 rpm for 5 minutes. The cell pellet was washed with sterile 1X PBS and cells were resuspended in fresh medium.

### Establishing radioresistant A 549 cell line

To discover and investigate mechanisms of radioresistance in NSCLC, we successfully established lung cancer cell model system with acquired radioresistance. We have developed an in-house A549 radioresistant cell line by serially irradiating with 4 Gy of X-ray radiation every second week to a total cumulative dose of 80 Gy using X-RAD 320 cell irradiator (Precision X-ray, Inc., North Branford, CT). During each experiment, growing cells were always treated with required dose of radiation and its radioresistance was regularly checked.

### Knockout of caveolin-1 by CRISPR-Cas9 mediated gene editing

Primer to generate CAV-1 targeting crRNA (AGTGTACGACGCGCACACCA)was synthesized (Integrated DNA Technologies), phosphorylated using PNK kinase (New England Biolabs) and cloned into pSpCas9(BB)-2A Puro (PX459) V2.0 (a gift from Feng Zhang, MIT, Cambridge, MA, USA; Addgene plasmid #62988) [25] to generate pTJK387. A549 cells were cultured and 105 cells are seeded into 6 well plates and transfected with pTJK387 using Lipofectamine 2000 (ThermoFisher Scientific). After 24 hours, cells were puromycin selected for 48 hours prior to being plated into 96 well plates at a concentration of 0.5 cell/ well to isolate single cell clones. Single cell clones were subsequently expanded and screened by Western Blot assay to identify Caveolin-1 knockout cell lines. The *CAV-1* gene expression construct was generated by PCR amplification (*CAV1*α: GCTAGCCACCATGTCTGGGGGCAAATACG and GTCGACTTATATTTCTTTCTGCAAGTTGATG and cloned into GFP-marked lentivector pWCC43 [26] using Nhe1-Sal1. All constructs were validated by sequencing. These single cell clones were expanded and harvested. Protein estimation by Western Blot assay was done to confirm the outcome of genome editing (Caveolin-1 knockout) with CRISPR-Cas9 system.

### Microarray processing and hybridization

U133 plus 2 microarray landscapes 1,300,000 unique oligonucleotides, which comprehend 47,000 transcripts and variants, and characterizes approx. 39,000 human genes. Preparation of cRNA, hybridization, and scanning of microarrays were achieved according to the Affymetrix protocol. In brief, 5 μg of total RNA was transformed into double-stranded cDNA by reverse transcription. Biotin-labeled cRNA was generated by converting the cDNA sample using a BioArray High Yield RNA Transcript labeling kit (Enzo Diagnostics). Labeled cRNA was hybridized to the Affymetrix U133 Plus 2.0 GeneChip while revolving at 60 rpm for 16 h at 45°C. After hybridization, the microarray was washed using the Affymetrix Fluidics Station according to the manufacturer’s protocol. Subsequently, chip was scanned in an Affymetrix 3000 7G scanner.

### Normalization, unsupervised, and supervised analysis

Data analysis was performed using oligo package in R. Raw data (.CEL) files were imported. The data were normalized using the Robust Multi-Array Average (RMA) algorithm [27]. LmFit of limma package was used to evaluate for differentially expressed genes with a p-value of <0.05. For comparison between samples without replicate, the differential expression genes are only identified with a fold-of-change (FC>1). Unsupervised analysis was performed using the Euclidian distance method of hierarchical clustering on all samples [28]. The differentially expressed genes were grouped into pathway using (Ingenuity Pathways Analysis) IPA and the expression of genes in each pathway are plotted as heatmap using Complex Heatmap R packages.

### Development of A549 cell line-based xenograft model

The study was conducted in accordance with ethical principles derived from guidelines that included the Declaration of Helsinki, 40 as well as following all relevant local requirements from University of Maryland. After the animal protocol approval from the Office of Animal Welfare Assurance (OAWA) University of Maryland School of Medicine (IACUC Protocol No: #0518008), we developed an orthotopic mouse model of Non-Small Cell Lung Cancer (NSCLC). We adopted a trans bronchial approach for injecting cancer cells in lung of 4 weeks old 15 athymic nude female BALB/c mice. The animal was anesthetized using isoflurane. A trans bronchial approach for injecting cancer cells was being tested in which. The nude mouse was positioned supine recumbent with the head elevated 90 degrees using a rubber band attached to the front upper teeth. The tongue was laterally pulled using a Mosquito-pean forceps which raised the lower jaw and exposed the glottis. The glottis and vocal cords were then easy to visualize using a surgical microscope with 10 magnified vision in a fixed position. Under direct visualization of the vocal cords using a surgical microscope, sterile, disposable 23-gauge 2.5-cm blunt-tip slightly curved metal catheter was introduced into the trachea. The entire length of the catheter was advanced till it reaches the lower lobe for injection. A total volume of 50ul was injected into the right lung (25ul A549 lung cancer cells + 25ul of Matrigel). Total procedure time, including anesthesia, was 15 to 20 minutes. After instillation, the mouse was given 100% oxygen, and the head was kept elevated at a 45-degree angle until recovery and then returned to the cages. For the cone-beam CT for Lung Tumor Imaging: once the rodent was anesthetized, it was placed stomach down onto the animal platform of the small animal radiation research platform (SARRP) and secured in place. The nose cone of the isoflurane system was placed over the nose and mouth of the rodent. The thoracic region of the rodent was imaged using the SARRP’s onboard CT scanner. The SARRP software was reconstruct a 3D image of the region, and the lung tumor was identified.

### Irradiation procedure and clonogenic assays

24 hours prior to irradiation, media was removed from the cell flask and rinsed with PBS. Cells were trypsinized and resuspended with media in a centrifuge tube. The cells were then centrifuged to cause pelleting. Supernatant was removed and cells were resuspended with fresh media. Cells were counted with equal proportion solution and trypan blue (BIORAD) in a TC-20 cell counter (BIORAD). 250 cells were seeded into each well of a 6-well plate (Corning), according to previously established plating efficiencies. At time of irradiation, all test groups were brought to Maryland University’s research irradiator. Doses of 2, 4, 6, and 8 Gy were administered to corresponding test groups.

Following irradiation, media was replaced for all trial groups. After an incubation period of ~12 days the media was removed and rinsed with PBS. 2ml of staining solution (86.3% dH2O, 10% PBS, 2.7% Formaldehyde, 1% Methanol, 0.05% solid Crystal Violet (Potts Lab Protocol)) was added to each well for 30min. The staining solution was discarded, and wells were rinsed with PBS to remove excess stain. Plates were then inverted and allowed to dry overnight. Colonies consisting of 50 cells or greater were counted via the ProtocCol3 (Synbiosis) for later statistical analysis.

### Immunohistochemistry (IHC) of human and mouse lung cancer tissues

IHC staining of lung tumor tissue was performed using rabbit anti-human active caveolin-1 antibodies as described earlier [28, 29] on the mouse model tumors and on 20 de-identified human lung carcinomas provided by the University of Maryland School of Medicine Center for Innovative Biomedical Resources Pathology Biorepository Shared Service, Maryland after an IRB approval from University of Maryland School of Medicine. Briefly, the lung tumors tissues were embedded in paraffin and sectioned at 2-4-μm thickness. Staining was performed using the kit (Abcam, MA, USA) following the manufacturer’s standard protocol (Vector Laboratories, Burlingame, CA). The tissue slides were blocked with 2.5% normal horse serum for 10 min. Samples were then incubated with rabbit anti-human active caveolin-1 antibody (dilution 1:50), for 12 hours at 4°C. Following thorough wash, the tissue slides were incubated with anti-rabbit IgG HRP secondary antibody for 10 minutes. The slides were then stained with 3,3′-diaminobenzidine (DAB) (Vector Laboratories) and counterstained with hematoxylin (Vector Laboratories), dehydrated, treated with xylene, and mounted. All slides were examined, pictures were taken using an Olympus BX41 microscope (Olympus America, Melville, NY).

### Optimization of ELISA assay to measure caveolin-1 in blood

The ELISA assay for caveolin-1 detection was performed using MyBiosource cat # MBS761759 ELISA kit. In brief, assay was performed in 96 well plate, standard range was used from 2500pg to 39.063pg/ml. sample wells and blank well position with proper dilutions was carefully labeled. Pure recombinant-caveolin-1 protein was used as standard. Further, 0.1 ml of sample was pipetted (Mouse serum, plasma) into test sample wells and sealed the plate with a cover and plate was incubated at 37°C for 90 minutes. After incubation plate content was discarded and plate was washed 2 times then, immediately 0.1 ml Biotin-labeled antibody working solution was added into each well. Further, plate was sealed and incubated at 37°C for 60 min. Plate was washed 3 times with wash buffer (350 ul) and, 0.1 ml of HRP-Streptavidin Conjugate (SABC) working solution was added into each well and plate and incubated at 37°C for 30 minutes. Plate was washed 5 times with wash buffer (350 ul) Subsequently, 90μl of TMB substrate was added and plate was incubated at 37°C in dark for 15min. The reaction was stopped by adding 50μl stop solution absorbance was recorded at 450 nm in microplate reader immediately after adding the stop solution and data was plotted.

### Cell lysate preparation and western blot analysis

Cells were grown to 80–90% confluency and harvested, and cellular proteins were extracted with lysis buffer [40 mM HEPES-NaOH (pH 7.4), 1% NP40, 0.5% sodium deoxycholate, 0.1% SDS,150 mM NaCl] containing 1 X cocktail of protease inhibitors (Roche, Indianapolis, IN). Protein was estimated by BCA protein estimation kit (ThermoFisher Scientific) separated on a 12.5% SDS-polyacrylamide gel and proteins electroblotted to nitrocellulose membranes (Bio-Rad, CA). After blocking with 5% nonfat dry milk and 0.1% Tween 20 in Tris-buffered saline, membranes were incubated at 4°C for 16 h with caveolin-1 anti-mouse polyclonal antibodies (1:5000) (Cell Signaling, CA). The membranes then incubated with peroxidase-labeled secondary antibodies and (Amersham Pharmacia, Piscataway, NJ) developed by Super Signal chemiluminescence substrate (Pierce, Rockford, IL). Actin protein levels were used as a control for adequacy of equal protein loading.

### Cancer genome data analysis

TCGA cancer genome data of Lung Cancer Adenocarcinoma and Squamous Cell Carcinoma patients was downloaded from The Cancer Genome Atlas (TCGA) database (https://portal.gdc.cancer.gov/). cBioPortal platform (www.cbioportal.org) from Memorial Sloan Kettering was used for analyzing multi-dimensional cancer genomics and clinical data [30]. Further, using cBioPortal bioinformatic tools we analyzed percent of cav-1 gene alteration (amplification, deletion and nonsense mutation), and mRNA regulation in lung cancer patient genomes. The survival plots were generated by cBioPortal tools using appropriate clinicopathological criteria, and calculated disease-free survival, overall survival.

## Results

### Caveolin-1 expression is upregulated in A549 radiation resistant cells

**Fig 1:**
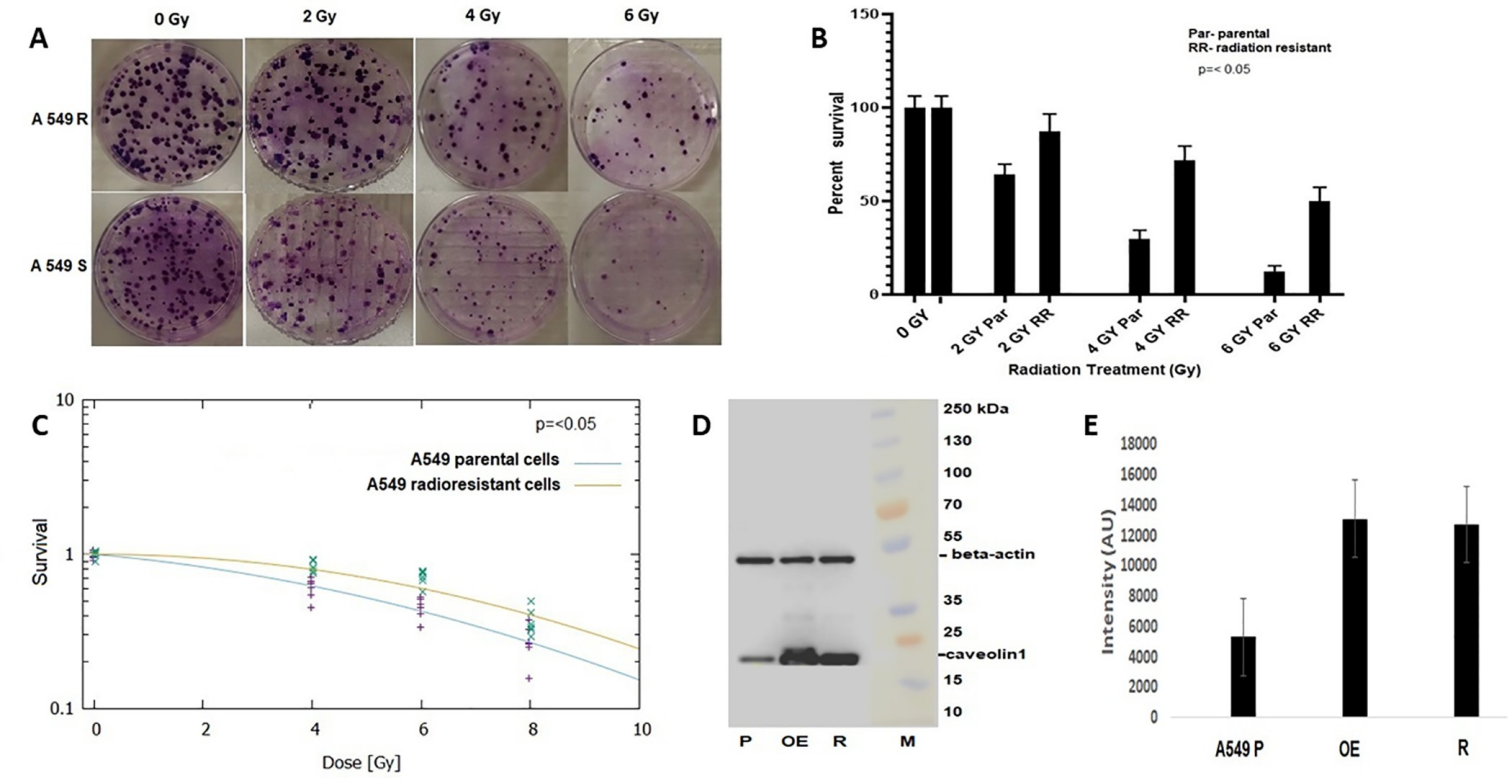
Over expression of caveolin-1 in radioresistant A549 lung cancer cells. **(A)** Clonogenic survival assay of parental and radio resistant A549 human lung cancer cell treated with 2, 4 and 6 Gy of radiation. (**B**), Percent survival of A549 parental and radioresistant cells treated with 2, 4 and 6 Gy of radiation. (**C**), Survival curve of A549 parental (blue) and radioresistant (Red) cells representing absorbed radiation dose and survival fraction using Linear-Quadratic Model. (**D**), Immunoblot analysis of Caveolin-1 (21 kDa) in the cell lysate of A549 parental (lane S), radio resistant (lane R), and CAV-1 overexpressed cells (lane OE).

To investigate molecular mechanism of radioresistance in NSCLC, we have established lung cancer cell model system with acquired radioresistance. We developed an A549 radioresistant cell line by serially irradiating with 4 Gy of X-ray radiation every second week to a total cumulative dose of 80 Gy. Clonogenic (Fig 1B), and MTT assay showed enhanced survival of A549 resistant cells and acquired radioresistance at higher doses of radiation (Fig 1B). The data has shown that cells acquired the ability to survive and tolerate much higher dose of radiation as compared to parental control cells (Fig 1A–1C). Parental A549, and radioresistant cells were processed for cell lysate preparation and equal concentration of proteins was resolved on SDS-PAGE and transferred onto nitrocellulose membrane for immunoblot analysis. We observed that caveolin-1 (21 kDa) was 2.5-fold upregulated in A549 radioresistant cells as compared to radiosensitive parental control (Fig 1D, lane 3; Fig 1E).

### Radioresistant A549 cells revert to radiosensitive by knocking out *CAV-1* gene and its overexpression induces radioresistance in parental A549 cells

**Fig 2:**
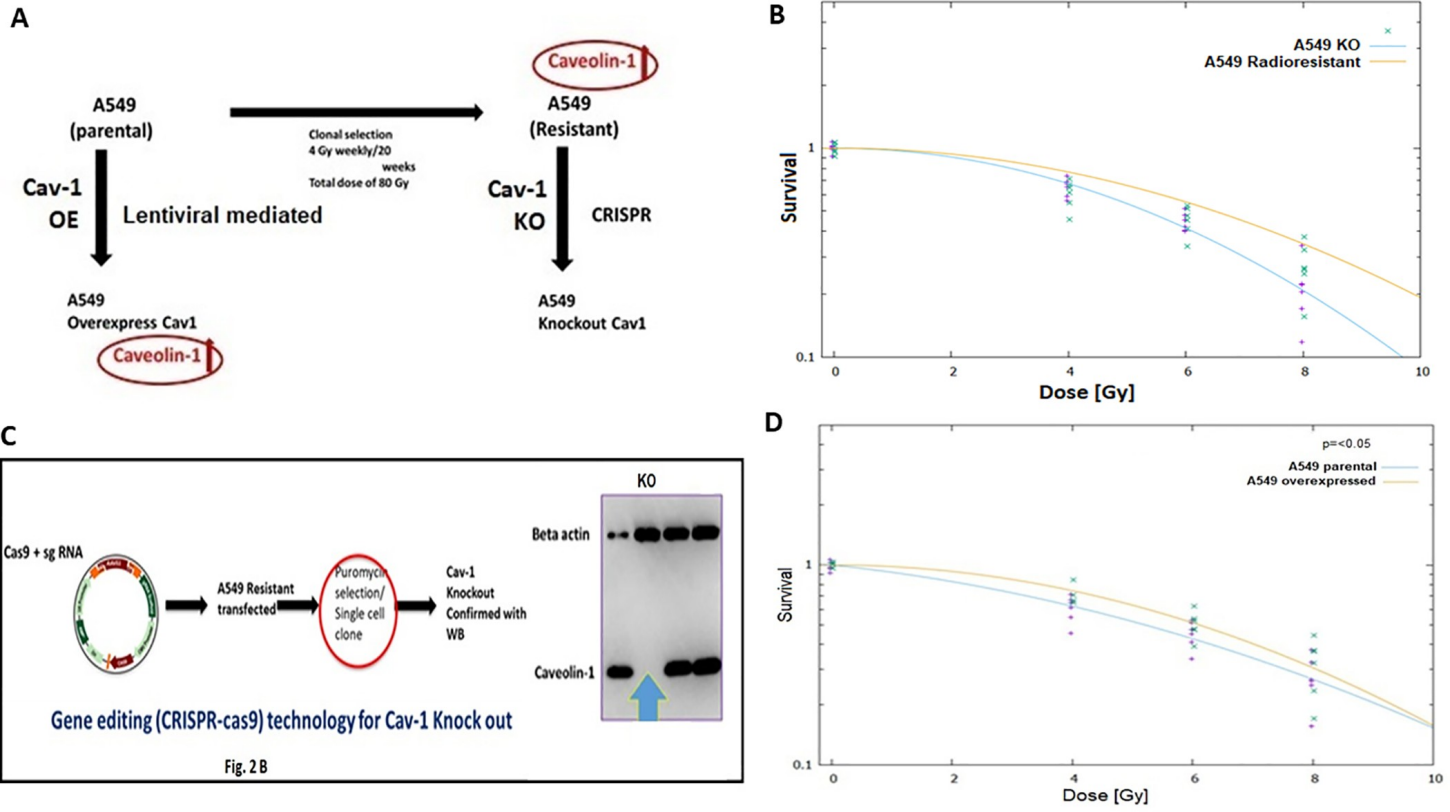
**(A)** Knock out of *CAV-1* gene by CRISPR CAS9 and effect of its overexpression in radioresistant A549 lung cancer cells. (A) Schematic diagram of generating radioresistant, CAV-1 KO and CAV-1 overexpressing A549 cells from parental A549 cells. (**B**), Survival curve of A549 radioresistant (Red) and CAV-1 KO cells (Blue), representing absorbed radiation dose and survival fraction using Linear-Quadratic Model. (**C**), Schematic diagram of generating CAV-1 knockout and CAV-1 overexpressing A549 cells by CRISPR-Cas9 gene editing technology. (**D**), Survival curve of A549 parental (Blue), and CAV-1 overexpressed cells (Red), representing absorbed radiation dose and survival fraction using Linear-Quadratic Model.

To determine whether *CAV-1* functionally contributed to radio-resistance we used CRISPR-Cas9 to generate *CAV-1* knockout cells. Briefly, we designed sgRNA targeting *CAV1* sgRNA1 PX459 to obtain the KO clone that inhibit caveolin-1 protein production (Fig 2B, lane 2) and screened single cell clone by western blot analysis to identify knockout cell lines (Fig 2B). The data presented in immunoblot showed CAV*-1* gene was successfully deleted in caveolin-1 knock out (KO) A549 cells (Fig 2B). We also observed that A549 resistant cells demonstrated radio-resistance and could tolerate elevated dose of radiation (Fig 2C), and *CAV-1* KO cells demonstrated increased sensitivity to radiation (Fig 2C). This demonstrated that knocking out of *CAV-1* reverses the acquired resistance. In addition, CAV-1 overexpressing cells also tolerated higher dose of radiation (Fig 2D) Conversely, lentivirus mediated overexpression of *CAV-1* in parental A 549 cells enhanced radioresistance and exhibited radio-resistance phenotypes.

### Microarray expression analysis of radio resistant A549 cells exhibit upregulation of DNA repair genes, antiapoptotic and cancer stem cells markers

**Fig 3:**
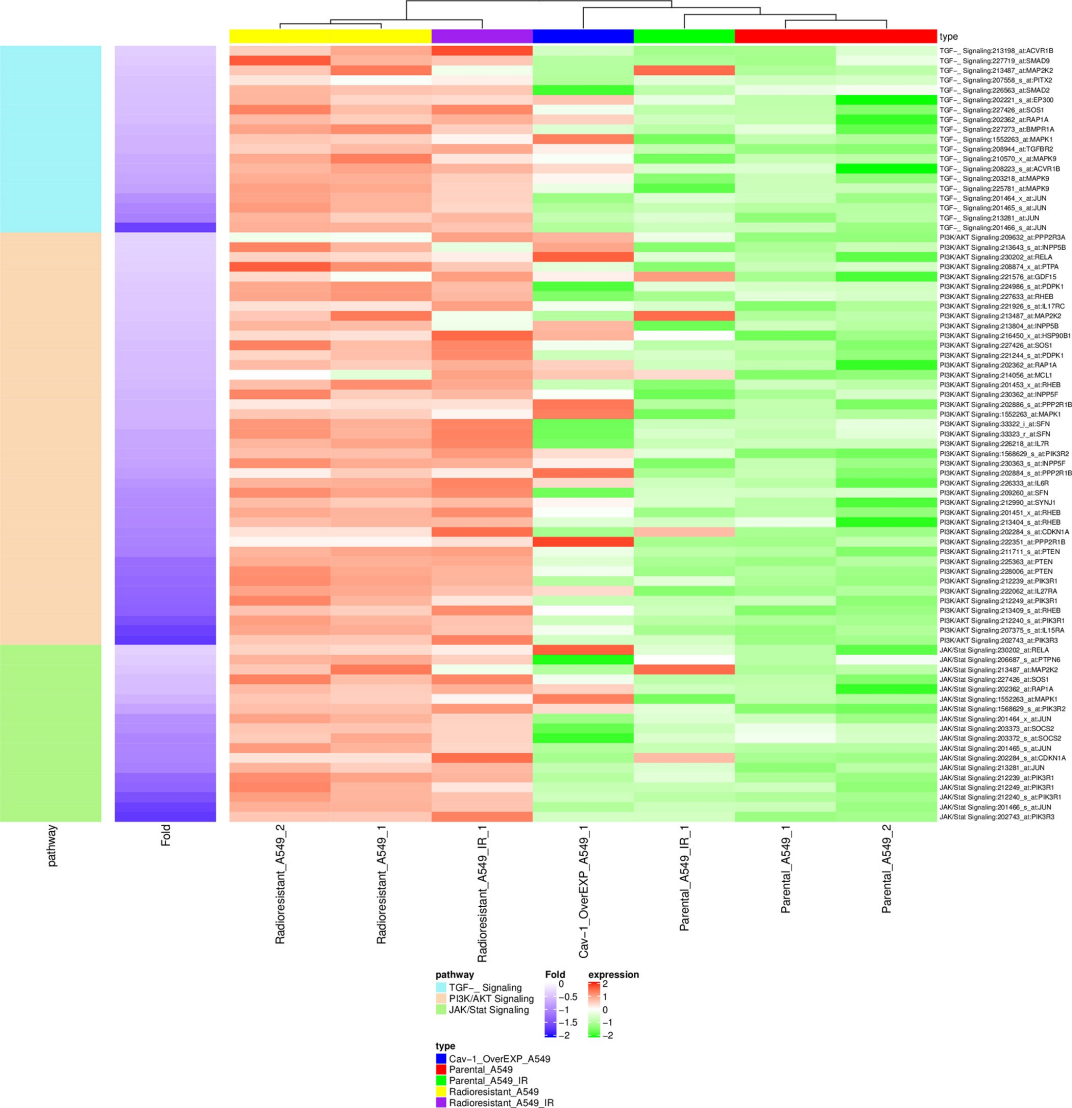
Heatmap analysis of gene expression profiling of parental A549, radioresistant and *CAV-1* overexpressed cells.

**Fig 4:**
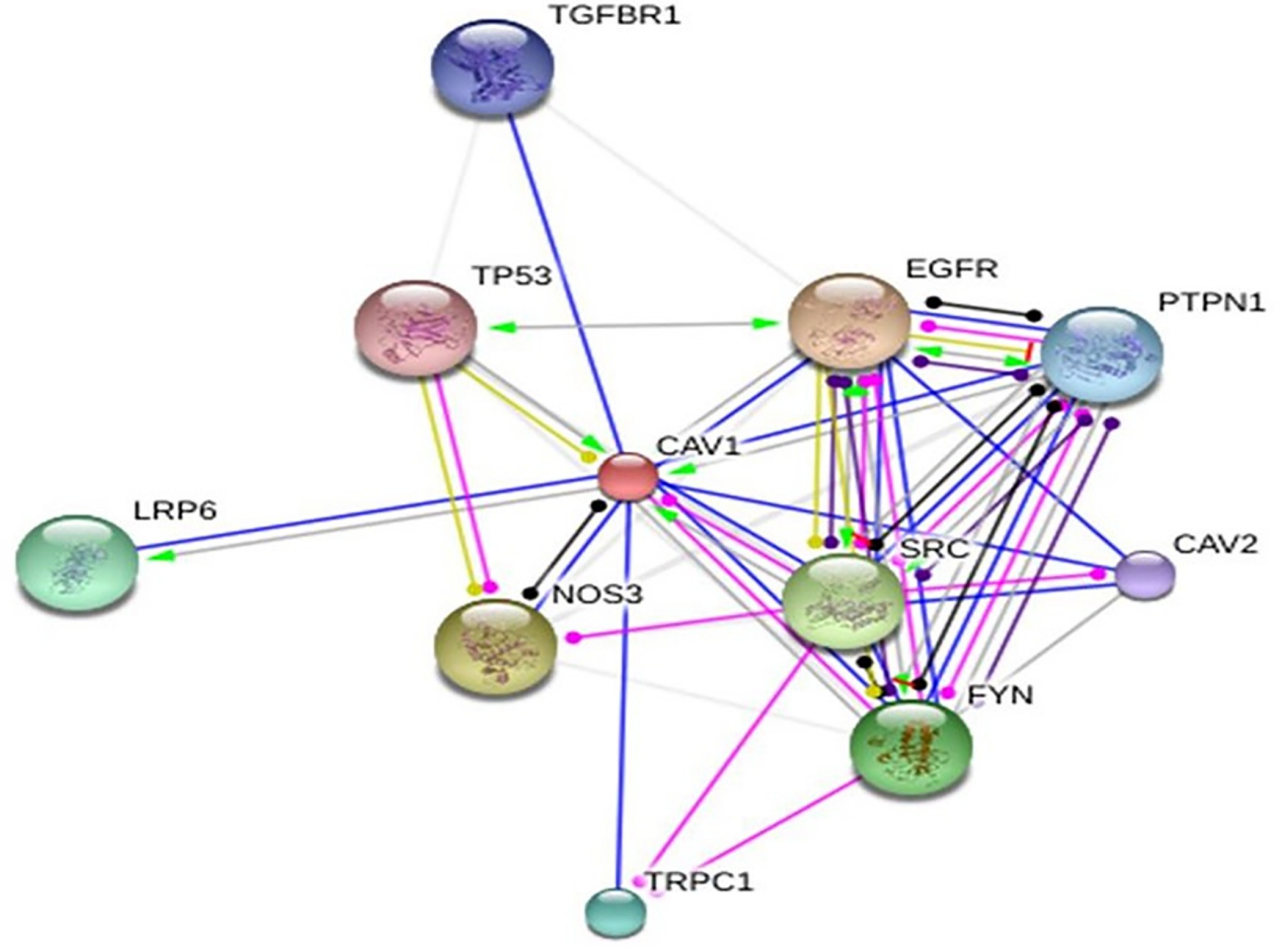
Protein-protein interaction network of human caveolin-1 in A549 radioresistant cells. Network was constructed using STRING 10.0 database. The network shows interaction between Caveolin-1 and other important interacting protein based on experiment, co-expression, text mining & co-expression with 0.007 confidence score as the analysis parameter.

Comparative gene expression profiling of A549 parental control, radioresistant, *CAV-1* knockout vs *CAV-1* overexpressed cells was carried out to examine the effect of *CAV-1* manipulation on gene expression pattern to identify genes whose expression correlate with radiation sensitivity vs radiation resistant phenotypes and in their phenotypic behavior. Human Genome microarray analysis revealed distinctive gene expression profile of sequential gene response to radiation in these cells. We accomplished global expression analysis of 47,000 transcripts and variants. Unsupervised hierarchical clustering of gene expression analysis has revealed that the A549 radioresistant cells, and *CAV1* overexpressed cells demonstrated different gene expression pattern compared to A549 parental control (Fig 3). In order to gain significant insight in gene expression, and fold changes, we had set up cut off value of >2.1 Fold up and <-2.1 fold down regulation. Based on our cut off filter we observed 700 genes upregulated in A549 radiation resistant cells, as compared to parental control. We also observed 1191 genes upregulated in parental control (sensitive). Bioinformatic analysis exhibited significant differences in gene expression pattern in radiation resistant, *CAV-1* KO and *CAV-1* over expressed A549 cells. Interestingly, the data have shown several fold upregulation of DNA repair genes like *RAD51b*, *RAD 18* and *SOX2* cancer stem cell markers, *MUC5AC* and *TGFBR2* in radiation resistant and caveolin-1, overexpressed A549 lung cancer cells (Table 1). We have also observed down regulation of anti-apoptotic genes *BCL2* family apoptosis regulator *BOK*, and *BCL2A1* in radiation resistant and caveolin-1 overexpressed cells (Table 1). In addition, the Ingenuity Pathway Analysis has shown upregulation of *BRCA1*, *NER*, *TGFB* and JAK/*STAT* pathways in radiation resistant A549 cells and BRCA1 and NER repair pathways are predominantly involved in DNA damage and response following radiation damage and cisplatin-based chemotherapy (S1 Fig). The bioinformatic analysis employing pathway database have also delineated upregulation of *TGFBR2-SMAD* and *JAK-STAT* pathways which might be also associated with caveolin-1 induced radio-resistance. Based on gene expression data we also created neighboring protein-protein interaction network which showed strong interaction of *CAV-1* with *EGFR*, *TGFBR1*, *TP53* and *CAV-2* (Fig 4). Thus, microarray gene expression data analysis further corroborated interaction of caveolin-1 protein with TGFB and CAV-2. We envisage that caveolin-1 also cross talk to EGFR and TGFB which could be involved in radio-resistance.

**Table 1:** Comparative gene expression (fold changes) profile analysis of genes in A549 parental, radioresistant, CAV-1 overexpressed and CAV-1 KO cells.

Gene Name	Gene symbol	A549 Control Cells (P)	Fold up in Rad Resistant	Fold up in CAV1 over expressed	Fold change in CAV1 Knock out
**RAD51 recombinase**	**RAD51B**	**1**	**2.269945788**	**8.944487092**	**1.00071696**
**RAD18, E3 ubiquitin protein ligase**	**RAD18**	**1**	**1.968225027**	**7.796698762**	**1.139976538**
**Matrix metallopeptidase 16**	**MMP16**	**1**	**16.86781089**	**6.824157022**	**-1.196954462**
**SRY-box 2**	**SOX2**	**1**	**5.069396975**	**8.369084986**	**1.064976283**
**Cell migration inducing hyaluronidase 1**	**CEMIP**	**1**	**12.36222998**	**2.268101652**	**-1.946942031**
**ADAM metallopeptidase domain 12**	**ADAM12**	**1**	**4.513203089**	**7.104283392**	**-2.153090703**
**Mucin 5AC, oligomeric mucus/gel-forming**	**MUC5AC**	**1**	**1.691245857**	**9.568691864**	**-1.92371853**
**Toll like receptor 4**	**TLR4**	**1**	**5.143265651**	**5.735514667**	**-1.127569265**
**Laminin subunit gamma 2**	**LAMC2**	**1**	**3.578967676**	**5.638541461**	**-1.134337609**
**Epidermal growth factor receptor**	**EGFR**	**1**	**1.988879257**	**-**	**-**
**TNF receptor superfamily member**	**TNFRSF**	**1**	**1.844388436**	**2.074485879**	**-1.563885232**

### Immunohistochemical analysis of caveolin-1 expression in human lung cancer tissues

**Fig 5:**
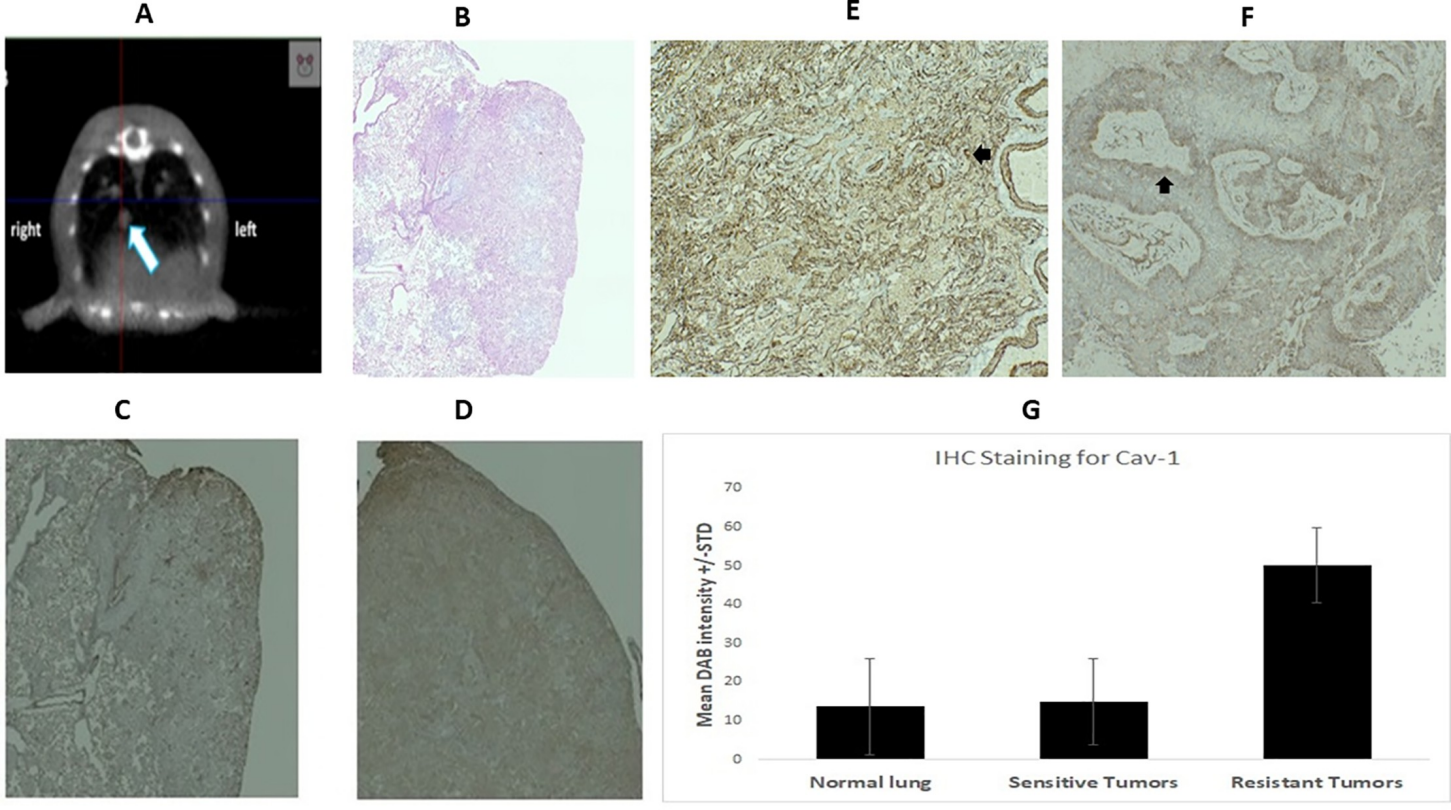
*In vivo* studies of radioresistant lung cancer in nude BALB/c mice and its histopathological characterization. **(A),** Orthotopic mouse model of human A549 lung cancer showed in Cone beam CT image following trans-bronchial inoculation of A549 NSCLC cells in the right lung of nude mice. White arrow shows tumor in lower lobe of right lung. (**B**), Hematoxylin and eosin (HE) staining of the orthotopic tumor in nude mice; (**C**), IHC staining of Caveolin-1 in in orthotopic lung tumor created by A549 parental cells. (**D**), IHC staining of Caveolin-1 in in orthotopic lung tumor created by A549 radio resistant cells. (**E**), IHC staining of Caveolin-1 in human adenocarcinoma cells; (**F**), IHC staining of Caveolin-1in human squamous cell carcinoma cells. (**G**), Quantification of IHC images of radiosensitive and radioresistant mouse tumor tissues.

We were able to generate orthotopic tumors in an animal model, with both radiosensitive (parental) and radioresistant A549 cells. We trans bronchially injected 20 ul of A549 parental cells (3X10^6^) with Matrigel in (1:1) into the lungs of 15 mice and, 15 mice with same number of radioresistant NSCLC cells (A549), in the right lung of nude mice. The lung tumor imaging was performed using SARRP’s (Small Animal Radiation Research Platform) onboard micro CT scanner. The SARRP software reconstructed a 3D image of the region, and the lung tumor was identified (Fig 5A). After 8 weeks, 15 animals were euthanized, blood was collected, and lung tissues were processed for H & E staining (Fig 5B). The data has shown that about 80% of animals injected with radioresistant cells developed radioresistant tumor as compared to 60% of animals injected with radiosensitive A549 cells. Immunohistochemical staining of caveolin-1 in mouse lung tissues also confirmed its higher expression in the radioresistant tumors compared to the sensitive (parental) cell line (Fig 5C, 5D and 5G). To further investigate expression of caveolin-1 in human lung cancer tissues, we performed immunohistochemistry analysis (IHC) in human lung tumor samples. 20 NSCLC human cancer samples were stained with the same protocol as the mice tumor. Interestingly, we were also able to stain caveolin-1 in human adenocarcinoma (Fig 5F), and human squamous cell carcinoma (Fig 5E), and 14 samples showed caveolin-1 positive staining The results showed heterogeneous staining of *CAV-1* was observed in lung cancer patients.

### Copy number amplification of *CAV-1* is associated with poor prognosis in lung cancer patients

**Fig 6:**
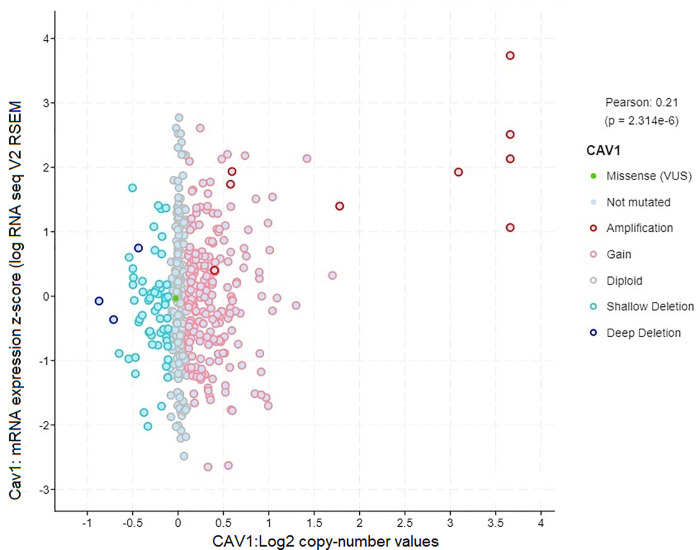
Genetic alteration profile of *CAV-1* in lung adenocarcinoma patient’s samples.

**Fig 7:**
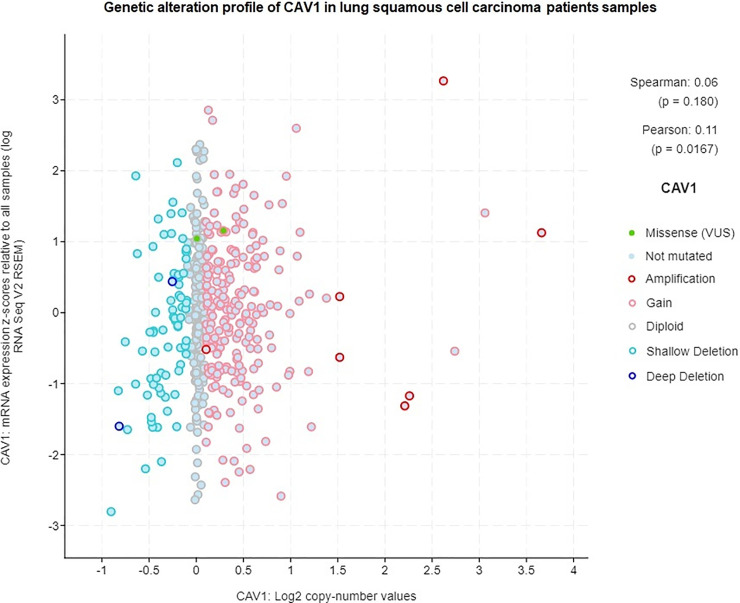
Genetic alteration profile of CAV-1 in lung squamous cell patient’s samples.

**Fig 8:**
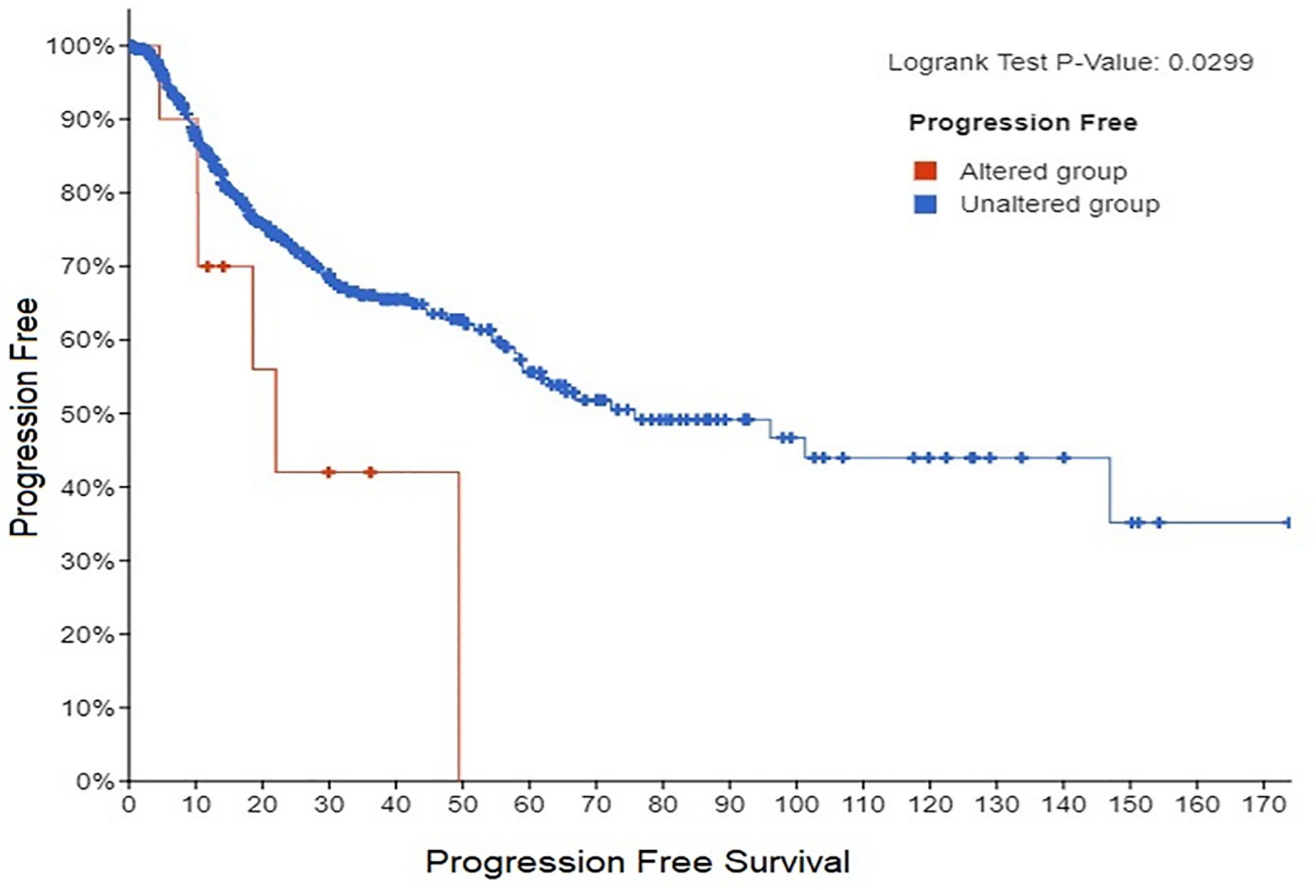
Kaplan-Meier plot of disease-free survival in *CAV-1* altered (Red), and *CAV-1* unaltered (Blue) group in lung cancer patients.

We analyzed lung cancer clinical data from TCGA PanCan NCI Cancer Genome Project using cBioPortal for Lung Cancer Genomics. We were able to identify 24 lung cancer patients (Adenocarcinoma and Squamous Cell Carcinoma) with altered *CAV-1* gene. Specifically, out of 24 lung cancer patients, majority of cancer them had *CAV-1* gene amplification linked with increased expression and copy number variation with significant p values (Figs 6 and 7). In addition, 5 cancer patients genome exhibited deep deletion in *CAV-1* gene with only 49% mRNA expression, and one patient had missense mutation with unknown significance (S2 Fig). Notably, out of 24 patients, 25% patients, were treated with radiation therapy (RT), and 9 patients had the disease recurrence. The mutual exclusivity analysis also showed missense mutations in two co-expressed *CAV-2* and *EGFR* genes with p value, 7.3e^-132^ and 0.05 respectively. These findings were further corroborated with the protein-protein interaction network in which *CAV-1* gene has shown interaction with *EGFR* and *CAV2* (Fig 4). Thus, the lung cancer genome and RNA seq data suggest that predominantly, there was significant amplification in *CAV-1* gene in lung cancer patients. Further, patient survival data also showed reduced progression free survival and poor prognosis of lung cancer patients carrying *CAV-1* mutation, and the disease-free survival among those patients was 50 months as compared to more than 179 months for patients without *CAV-1* genetic alteration (Fig 8).

### Caveolin1 as a biomarker for lung cancer aggression and radio-resistance

**Fig 9:**
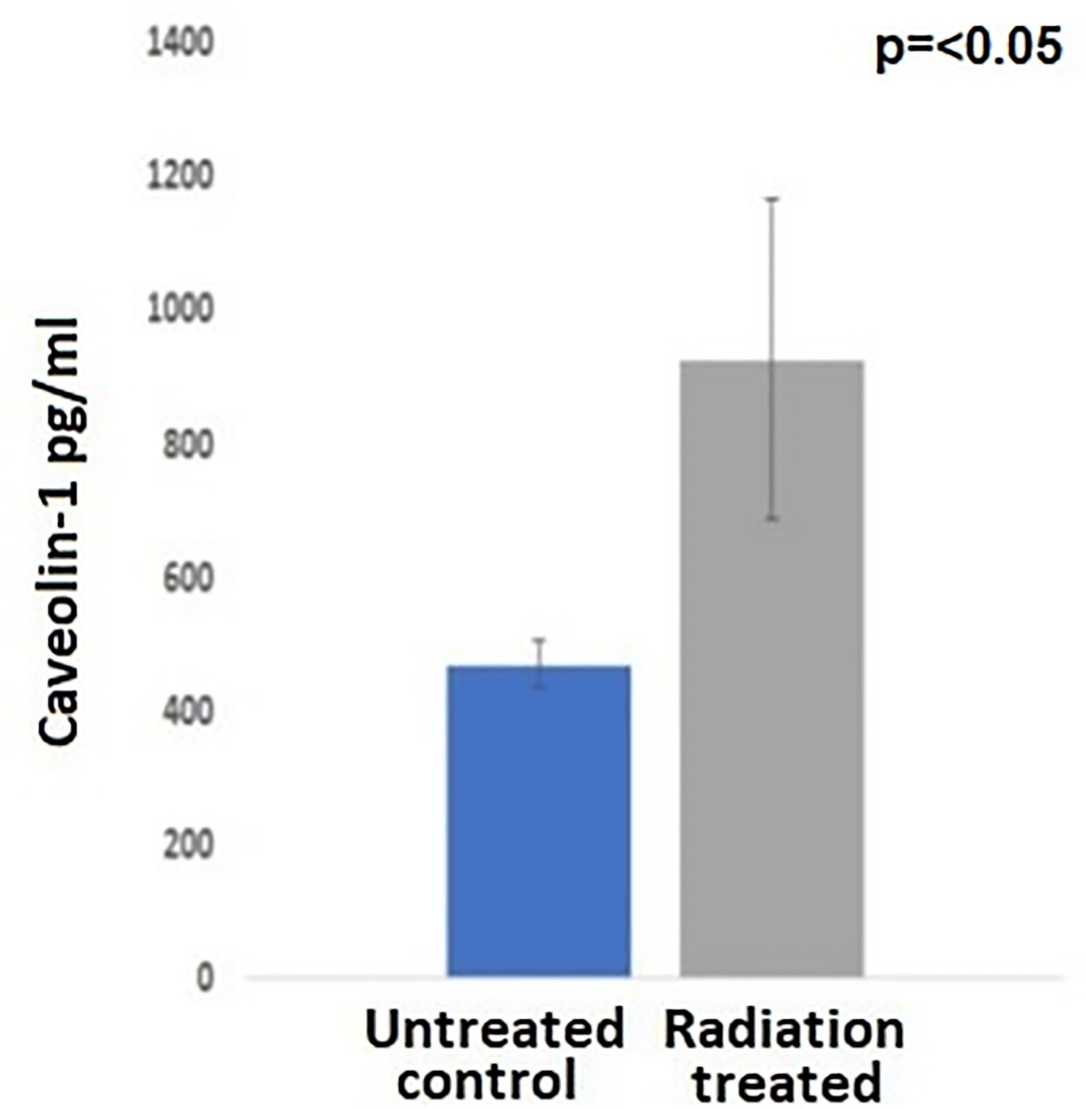
Optimization of ELISA assay in the blood of radiation treated, and untreated human lung cancer xenograft mouse model.

In order to examine the clinical potential of caveolin-1 as a liquid based biomarker for radio-resistance in lung cancer, we have made an attempt to demonstrate that caveolin-1 could be used as liquid based biomarker to predict radio-resistance in cancer patients. We created radiation resistant tumor by trans bronchial injection of radiation resistant A549 cells, and parental cells as control group. After treating animals with radiation, we euthanized animals and collected blood samples for ELISA assay. The data presented in Fig 9 demonstrate that animals developed radioresistant tumor had expressed high level of caveolin-1 almost two-fold higher as compared to parental control. Interestingly, higher level of caveolin-1 expression in lung tissues is also being released into circulatory system and it could be easily quantified by ELISA assay. This data is quite significant and caveolin-1 as liquid biomarker for radio-resistance in lung cancer may have clinical application. The results have shown that the median level of caveolin-1 (n = 15), was approx. 950 pg/ml almost two-fold higher in tumor bearing animals as compared to control animals (450 pg/ml) (Fig 9). Thus, the data demonstrated that caveolin-1 could be used as a blood biomarker for radio-resistance, and tumor progression in lung cancer patients.

## Discussion

Lung cancer is a considerable global public health problem and is associated with poor prognosis with overall survival after 5 years only 16% [1]. For locally advanced and non-resectable forms of NSCLC, radiotherapy and chemotherapy are standard of care and play an immensely important role in killing tumor cells. However, due to local recurrence cancer cells develop radio-resistance and patients do not respond to radiation therapy [9, 31]. Thus, there is an urgent need to develop better and urgent treatment options for effective radiation therapy. The in-depth analysis of molecular mechanisms involved in radio-resistance in non-small cell lung carcinoma is urgently needed to develop innovative approaches to ameliorate the efficacy of radiation and chemotherapy in cancer patients. In the present investigation, we have identified and characterized caveolin-1 as promising biomarker of radio-resistance and tumor progression in lung cancer [14].

In this study, we have characterized radiation resistant cell line model that we developed by exposing A549 parental cells to gradually higher doses of radiation to unravel molecular pathways that lead to radio-resistance. Based on experimental evidence presented in immunoblot analysis and clonogenic assay indicates that the upregulation of caveolin-1 in radiation resistant cells could be one of the radiation induced pathways activated in cancer cells exhibiting radio-resistance phenotype. In order to further explore the role of caveolin-1 in radio-resistance, we knocked down *CAV-1* gene by CRISPR-Cas9, and interestingly, the resulting knock out cells reverted to radiosensitive phenotype. These findings are consistent with other reports in which *CAV-1* silencing made pancreatic cancer cells sensitive to radiation and chemo treatment [21, 32]. In another study, elevated level of caveolin-1 in prostate cancer tumor vasculature has been linked to radio-resistance in MPR31-4 prostate cancer xenograft tumors, and its knock down reversed radio-resistance in those animals [23]. In recent report *CAV-1* has been linked to radiation and chemoresistance in cisplatin-resistant ovarian cancer cells [33, 34], Thus, based on our data we concluded that overexpression of caveolin-1 contributes to radio-resistance and tumor aggression in lung cancer (S3 Fig).

Further, to explore the genomic network of caveolin-1, comparative gene expression analysis of A549 parental control, *CAV-1* overexpressed, and *CAV-1* KO was performed. We found that the gene expression profile in radiation resistant and overexpressed cells was significantly different than the parental control (Fig 3). However, the *CAV-1* expression at mRNA level was changed at the threshold level, which could be due to its regulatory and oncogenic role. Specifically, the DNA repair genes like *RAD51B* and *RAD18*, cancer stem cell marker *SOX2* and antiapoptotic genes were significantly up regulated in radiation resistant cells. The expression data also showed 1.7-fold up regulation of *MUC5AC* in radiation resistant cells, and functionally it has been linked to focal adhesion kinase (*FAK*) phosphorylation, and lung cancer proliferation [35], and KRAS mutated aggressive lung cancer [36]. The high throughput Ingenuity Pathway Analysis was also performed, and we were able to identify upregulated Nuclear Excision Repair, *BRCA1* DNA repair pathways (S1 Fig). We could also identify *TGFB*, *PI3K-AKT* and JAK/*STAT* pathways in radiation resistant and caveolin-1 overexpressed cells (Fig 3). However, we also found upregulation of BCL2a1 gene in A549 radio-resistant cells which does not seems to be highly significant and currently we are validating in wet lab (Table 1). We have also found enhanced expression of caveolin-1 in lung tissues of animals injected with A549 radioresistant cells and these results were supported by caveolin-1 expression in human lung tissues which could be responsible radio-resistance [34].

The corollary findings have also shown enhanced NER pathway activation in lung cancer following treatment with DNA damaging agents [37, 38]. Interestingly, recent report also suggests its involvement of caveolin-1 in DNA damage and repair in breast cancer cells [39]. Further, the predicted protein-protein interaction network of *CAV-1* also revealed interaction among caveolin-1-EGFR-TGFBR-TP53, which clearly suggest a critical role of caveolin-1 in tumor progression, radiation and chemoresistance in lung cancer. Our experimental data also delineate that radiation induced oxidative stress aberrantly activate membrane bound caveolin-1, which interacts with EGFR and TGFB and activate further downstream signaling. Consequently, TGFB activates SMAD and caveolin-1 transport EGFR to the nucleus which transcribes target genes involved in DNA repair and radio-resistance. Recent reports have also suggested an oncogenic role of *CAV-1* in pancreatic, breast and ovarian cancers [14, 21, 23, 38]. Thus, the present data strongly suggest that caveolin-1 plays an immensely important role in radio-resistance in lung tumorigenesis. Currently, we are working on caveolin-1 induced resistance pathway and efforts are being made to precisely identify proteins involved in these pathways responsible for radioresistant phenotypes. Since, caveolin-1 is a membrane protein and we tried using shRNA-based silencing and the data was not promising. Therefore, currently we are trying to develop small molecule inhibitor against caveolin-1 based on the Y14 structure at N-terminal domain [14].

The comprehensive TCGA study of lung cancer patients analyzed by cBioPortal has revealed a very important clinical role of *CAV -1* in lung cancer aggression. The TCGA lung cancer genomic data analysis of lung cancer patients has shown *CAV-1* gene amplification in 16 lung cancer patients, and 5 had *CAV-1* gene deletion, and 3 had missense mutation in *CAV-1* gene (Figs 6 and 7) [40, 41]. Importantly, these patients with *CAV-1* alteration had worse prognosis, and significantly reduced progression free survival when compared with patients without *CAV-1* gene alteration (Fig 8). An important clinical finding also reported elevated *CAV-1* expression in 69 cases of brain metastasis from lung cancer (NSCLC), that instigated radioresistance and very poor outcome [42].Thus, TCGA clinical data clearly suggest that *CAV-1* amplification is linked to copy number variation which is involved in altering downstream oncogenic signaling pathways that are aberrantly dysregulated in lung cancer, and responsible for radio-resistance and tumor aggression [43, 44]. Our present investigation on the role of *CAV-1* in radio-resistance, and important clinical findings based on cancer genome data suggest that *CAV-1* gain of function mutation might be a driver mutation in lung cancer tumor aggression [41]. Moreover, we have also discovered caveolin-1 as a potential clinical biomarker for lung cancer which could be used in clinical setting to monitor patient’s response to radiation therapy and recurrence (Fig 9) [14]. Interestingly, the TCGA data on lung cancer patients also supported our hypothesis that *CAV1* gene deletion causes loss of functional caveolin-1 protein which is involved in cancer aggression and chemo and radio-resistance [21, 32], and caveolin-1 loss has also been linked to cancer aggression [45]. Further, gene amplification of *CAV1* by copy number variation has been linked to cancer aggression and metastasis. It is envisaged that multiple copies of the gene aberrantly regulates the oncogenic downstream signaling by genetic fix [46, 47].

We were not able to examine caveolin-1 expression in other cell lines, However, in TCGA data analysis we found CAV1 amplification in both lung cancer adenocarcinoma and squamous cell carcinoma and due to multicopy number variation caveolin-1 protein expression is elevated. Similarly, deep deletion caused truncation in caveolin-1 protein and as a result it exerted different phenotypic effect. Thus, TCGA data showed that modified CAV1 induced worst prognosis in lung cancer patients.

## Conclusion

In conclusion, we report that caveolin-1 plays an immensely important role in radio-resistance and cancer progression in lung adenocarcinoma, and caveolin-1 has the potential to be used as prognostic biomarker for radiation treatment response and tumor aggression in clinical setting for precision medicine.

## Supporting information

S1 Fig Upregulation of PI3K/AKT pathway in A549 radioresistant cancer cells and its comparison with A549 parental cancer cells. (PDF)

S2 Fig Oncoprint view of CAV1 genetic alteration in lung cancer patients. (JPG)

S3 Fig Cell proliferation assay of A549 radioresistant cancer cells and parental cancer cells. (JPG)

S4 Fig (JPG)
