# Correction: Role of caveolin-1 as a biomarker for radiation resistance and tumor aggression in lung cancer

**DOI:** 10.1371/journal.pone.0268256

**Published:** 2022-05-03

**Authors:** Dominic Leiser, Santanu Samanta, Josh Strauss, John Eley, Michael Creed, Tami Kingsbury, Paul N. Staats, Binny Bhandary, Minjie Chen, Tijana Dukic, Sanjit Roy, Javed Mahmood, Zeljko Vujaskovic, Hem D. Shukla

The authors are listed out of order. Please view the correct author order, affiliations, and citation here:

Dominic Leiser^1^, Santanu Samanta^1^, Josh Strauss^2^, John Eley^2^, Michael Creed^3^, Tami Kingsbury^3^, Paul N. Staats^4^, Binny Bhandary^1^, Minjie Chen^1^, Tijana Dukic^1^, Sanjit Roy^1^, Javed Mahmood^1^, Zeljko Vujaskovic^1^, Hem D. Shukla^1^

**1** Division of Translational Radiation Sciences (DTRS), Department of Radiation Oncology, University of Maryland School of Medicine, Baltimore, MD, United States of America, **2** Department of Radiation Oncology, School of Medicine, Vanderbilt University, Nashville, TN, United States of America, **3** Department of Physiology, University of Maryland School of Medicine, Baltimore, MD, United States of America, **4** Department of Pathology, University of Maryland, School of Medicine, Baltimore, MD, United States of America.

Leiser D, Samanta S, Strauss J, Eley J, Creed M, Kingsbury T, et al. (2021) Role of caveolin-1 as a biomarker for radiation resistance and tumor aggression in lung cancer. PLoS ONE 16(11): e0258951. https://doi.org/10.1371/journal.pone.0258951.
