# Supplementary figures and images for: Role of caveolin-1 as a biomarker for radiation resistance and tumor aggression in lung cancer
Source: PLoS One. 2021 Nov 11;16(11):e0258951. doi: 10.1371/journal.pone.0258951 (PMC8584669; doi:10.1371/journal.pone.0258951)

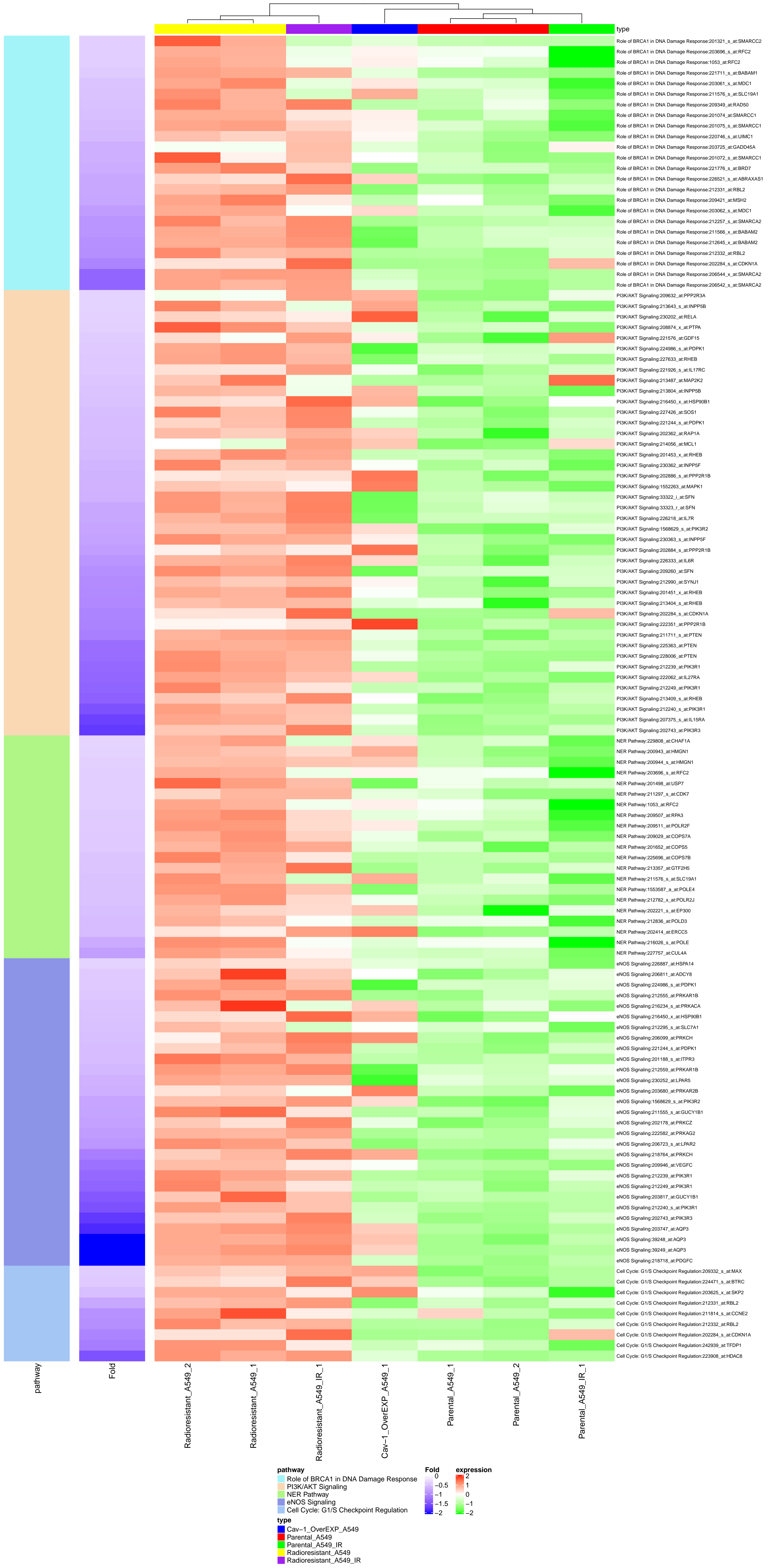

Supplement: S1 Fig — (PDF) [file pone.0258951.s001.pdf]

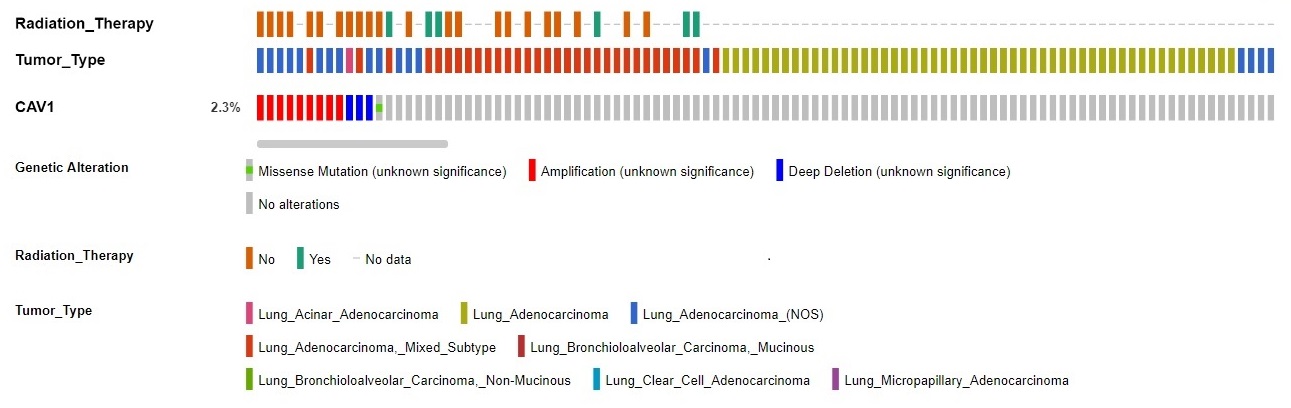

Supplement: S2 Fig — (JPG) [file pone.0258951.s002.jpg]

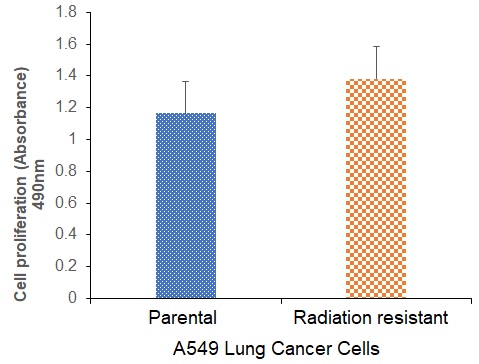

Supplement: S3 Fig — (JPG) [file pone.0258951.s003.jpg]

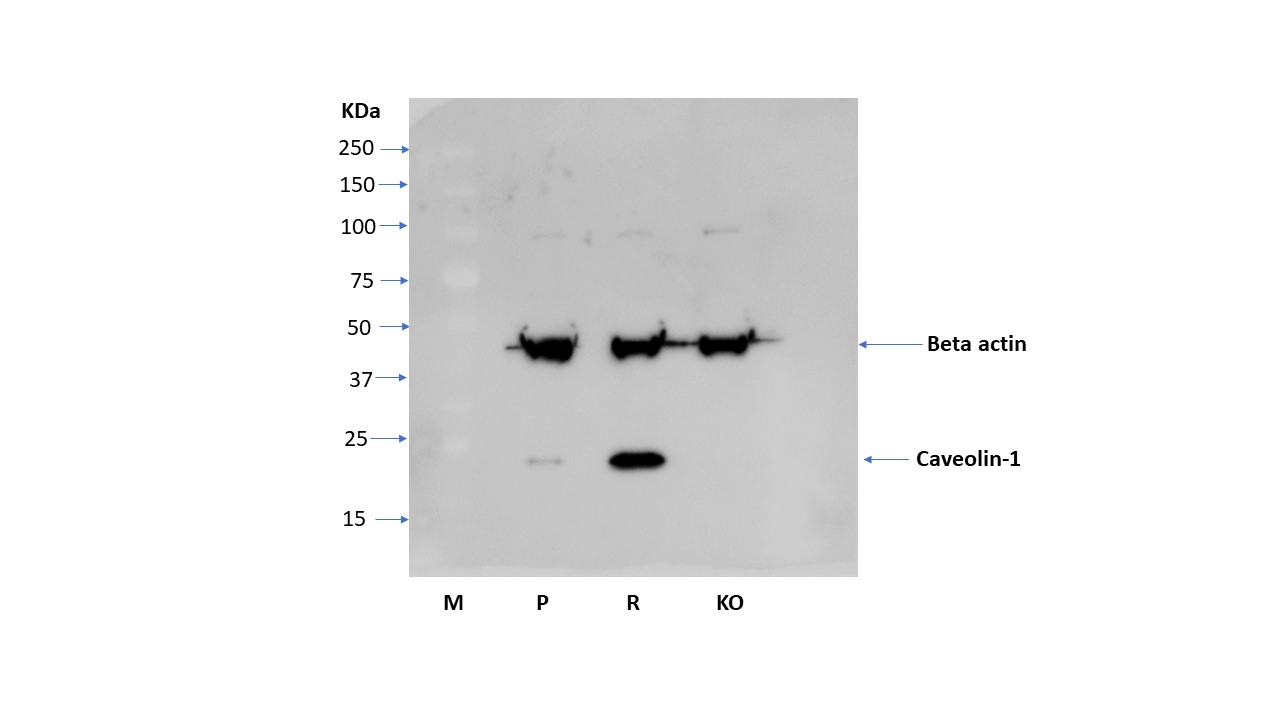

Supplement: S4 Fig — (JPG) [file pone.0258951.s004.jpg]
